# Photoelectrocatalytic Reduction of CO_2_ to Paraffin Using p-n Heterojunctions

**DOI:** 10.1016/j.isci.2019.100768

**Published:** 2019-12-12

**Authors:** Jinyuan Wang, Yongji Guan, Xiaogang Yu, Youzhi Cao, Jiazang Chen, Yilin Wang, Bin Hu, Huanwang Jing

**Affiliations:** 1 State Key Laboratory of Applied Organic Chemistry, College of Chemistry and Chemical Engineering, Lanzhou University, Lanzhou 730000, China; 2 State Key Laboratory of Coal Conversion, Institute of Coal Chemistry, Chinese Academy of Sciences, Taiyuan 030001, China; 3 Key Laboratory of Colloid, Interface and Chemical Thermodynamics, Chinese Academy of Sciences, Beijing 100190, China; 4 State Key Laboratory for Oxo Synthesis and Selective Oxidation, Lanzhou Institute of Chemical Physics, Chinese Academy of Sciences, Lanzhou 730000, China

**Keywords:** Catalysis, Electrochemical Materials Science, Materials Design

## Abstract

Nowadays, photoelectrocatalytic (PEC) reduction of CO_2_ represents a very promising solution for storing solar energy in value-added chemicals, but so far it has been hampered by the lack of highly efficient catalyst of photocathode. Enlightened by the Calvin cycle of plants, here we show that a series of three-dimensional C/N-doped heterojunctions of Zn_x_:Co_y_@Cu are successfully fabricated and applied as photocathodes in the PEC reduction of CO_2_ to generate paraffin product. These materials integrate semiconductors of p-type Co_3_O_4_ and n-type ZnO on Cu foam to construct fine heterojunctions with multiple active sites, which result in excellent C-C coupling control in reduction of CO_2_. The best catalyst of Zn_0.2_:Co_1_@Cu yields paraffin at a rate of 325 μg·h^−1^ under −0.4 V versus saturated calomel electrode without H_2_ release. The apparent quantum efficiency of PEC cell is up to 1.95%.

## Highlights

• Heterojunctions of Zn_x_:Co_y_@Cu are applied in photoelectrocatalytic reduction of CO_2_
• Multiple active sites result in excellent C-C coupling like natural photosynthesis
• Photoelectrocatalytic system can tolerate the higher voltage without H_2_ emission
• Paraffin product is reported for the first time in CO_2_ reduction

## Graphical Abstract

**Figure:**
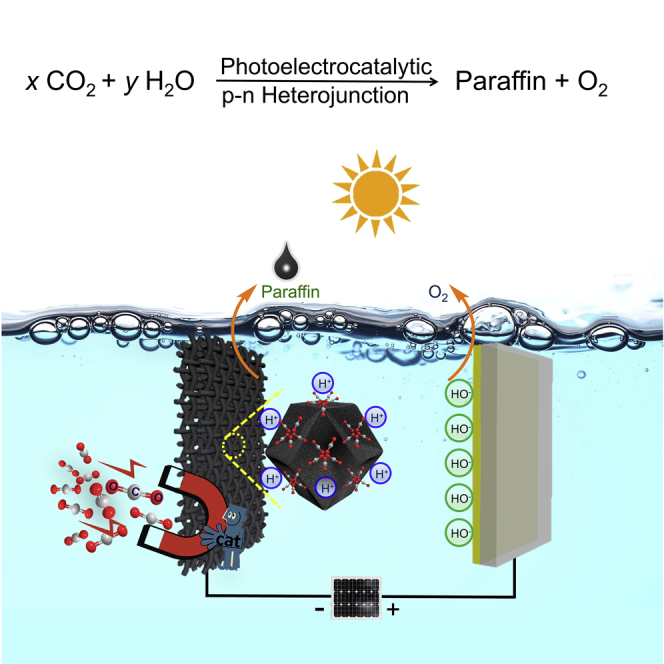

## Introduction

Solar energy as a clean, cheap, and sustainable energy source remains the final hope to human beings. Photosynthesis in plants and algae can efficiently utilize sunlight to convert CO_2_ and water to various organic compounds and O_2_ that feed organisms to complete the natural carbon cycle in the planet (Govindjee and Krogmann, 2004, Nürnberg et al., 2018). The photoelectrocatalytic (PEC) reduction of CO_2_ can be termed as *artificial photosynthesis*, which mimics natural photosynthesis and efficiently converts CO_2_ and H_2_O into hydrocarbons and O_2_, tackling both energy and global environmental problems (Halmann, 1978, Wang et al., 2019). Recently, semiconductors are commonly utilized as catalysts in PEC reduction of CO_2_, because they combine the advantages of both photocatalysis and electrocatalysis (EC) to promote the separation of photogenerated electron-holes leading to high solar conversion efficiency (Chang et al., 2019). However, their product distribution is usually limited to C1 and C2 compounds (CO, HCOOH, CH_3_OH, and C_2_H_5_OH, etc.) (Barton et al., 2008, Cardoso et al., 2018, Schreier et al., 2015, Shan et al., 2019). In contrast, the production of multi-carbon chemicals is much sought after as potential sustainable fuels, which represents a promising path toward establishing a carbon-neutral cycle (Loiudice et al., 2016, Zhuang et al., 2018). Unfortunately, it is difficult to generate multi-carbon chemicals using direct CO_2_ reduction owing to the low activity of the present catalysts in C-C coupling. This is currently the major scientific challenge in sustainable energy research.

Based on the knowledge of Calvin cycle (Bassham et al., 1954, Mao et al., 2004), we speculate that the photocathode of three-dimensional (3D) semiconductor heterojunction with multiple active sites is favorable for C-C coupling process in CO_2_ reduction. Recently, our groups introduced that constructing suitable semiconductor heterojunctions is a valuable strategy to improve their PEC performances (Xu et al., 2018) by enhancing the efficiency of separation of photogenerated electron-holes. Moreover, nitrogen-modified semiconductors show favorable performance in the absorption and activation of CO_2_ (Jia et al., 2017). In addition, metal oxides have been explored as efficient PEC catalysts. Among them, Co_3_O_4_ is an important p-type semiconductor with attractive photoelectric properties (Long et al., 2006, Tang et al., 2016); ZnO has been recognized as an excellent material for photocatalytic reactions (Liu et al., 2016, Yu et al., 2015), and Cu is confirmed in previous work as a unique metal that retains the significant faradaic yields of hydrocarbons and oxygenates (Lee et al., 2018, Lum and Ager, 2018, Shibata et al., 2008). As mentioned above, we choose Zn/Co-based zeolitic imidazolate frameworks (Zn_x_/Co_y_-ZIFs) as the substrates that were *in situ* assembled on Cu foam and led to Zn_x_:Co_y_@Cu through a calcined process.

Herein, we report that well-designed 3D C/N-doped heterojunctions of Zn_x_:Co_y_@Cu *in situ* integrated semiconductors of p-type Co_3_O_4_ with n-type ZnO on Cu foam are successfully used as photocathode in PEC cell for efficient solar-driven CO_2_ reduction. Notably, visible paraffin is produced through excellent C-C coupling control in our PEC cell of Zn_x_:Co_y_@Cu | KHCO_3_ | BiVO_4_. We reason that the possible mechanism of generating paraffin product arises from these ideal heterojunctions that ameliorate their abilities of harvesting solar light and enhance the separation efficiency of photogenerated electron-holes; the multiple active sites of catalyst result in a cooperative effect to realize high efficiency of C-C coupling and suppress H_2_ release. This is an important scientific discovery because this is the first tangible evidence uncovering chain propagation during PEC reduction of CO_2_ over semiconductor photocathode.

## Results and Discussion

### Preparation and Characterizations of Photocathodes

**Figure 1:**
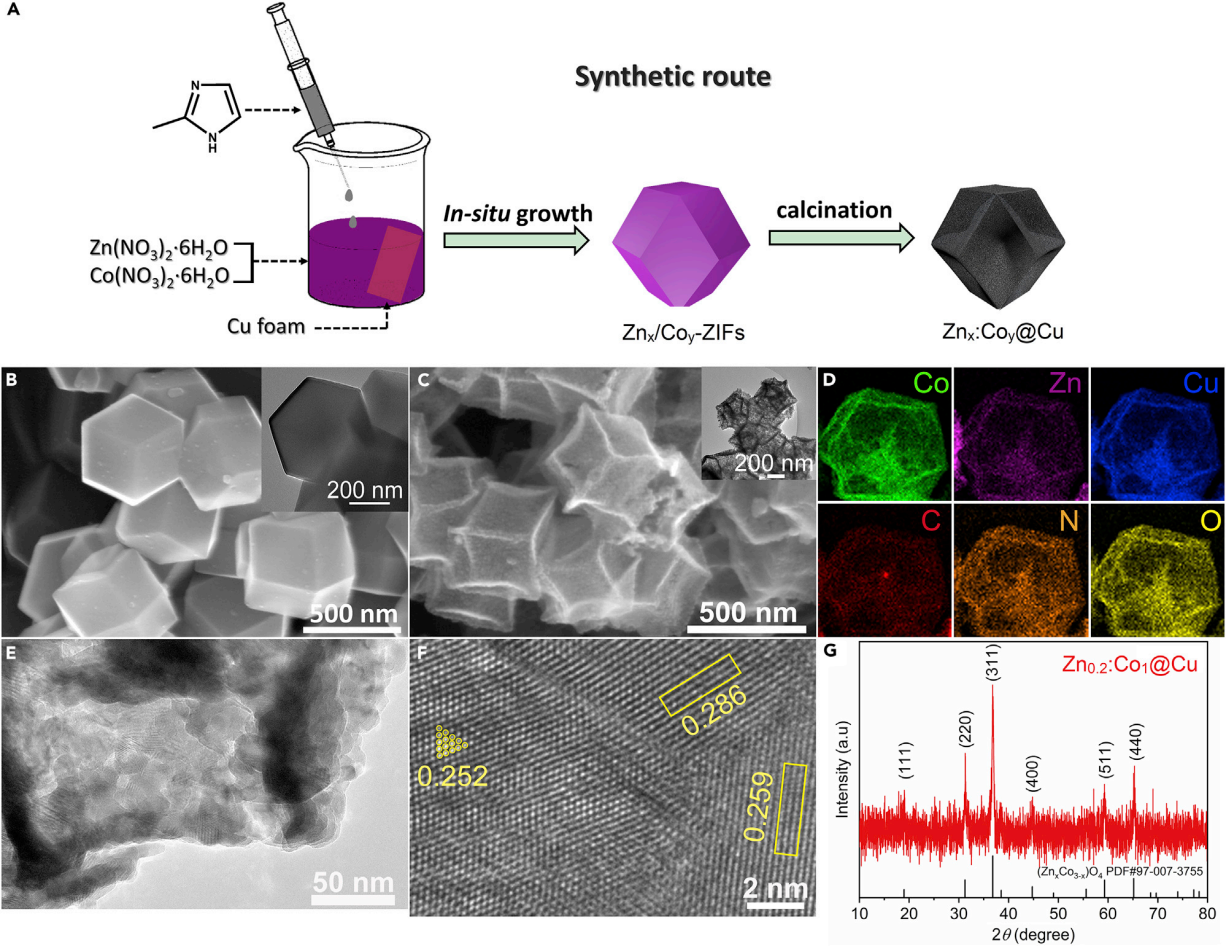
The Structure Characterization of Zn_0.2_:Co_1_@Cu (A) Schematic illustration of the fabrication procedures of Zn_x_:Co_y_@Cu photocathode. (B) SEM images of Zn_0.2_/Co_1_-ZIFs. (C) SEM images of Zn_0.2_:Co_1_@Cu. See also Figure S1. (D) Elemental mapping images of Zn_0.2_:Co_1_@Cu. (E) TEM image of Zn_0.2_:Co_1_@Cu. (F) HRTEM image of Zn_0.2_:Co_1_@Cu. See also Figure S4. (G) Corresponding X-ray diffraction patterns. See also Figures S2 and S3.

The fabrication procedures of Zn_x_:Co_y_@Cu are illustrated in Figure 1A, and the experimental details are summarized in the Transparent Methods. The precursors of Zn_x_/Co_y_-ZIFs are synthesized based on reported method (Banerjee et al., 2008, Wu et al., 2014); their morphology shows a regular dodecahedron structure (Figure 1B). In this work, the samples are prepared with different molar ratios of Zn(NO_3_)_2_·6H_2_O to Co(NO_3_)_2_·6H_2_O, and the actual ratios are determined by inductively coupled plasma-optical emission spectroscopy and listed in Table S1. We take the Zn_0.2_:Co_1_@Cu photocathode as the model catalyst for analyzing the morphology and structure. Scanning electron microscopy reveals an approximate diameter of 400-nm shrink hollow dodecahedron structure with sharp edges and rough surfaces (Figure 1C and Table S2). The X-ray diffraction patterns demonstrate spinel structure for Zn_x_Co_3-x_O_4_ (Figure 1G). High-resolution transmission electron microscopic (HRTEM) analysis indicates that the clear lattice fringes are related to the plane (220) of Co_3_O_4_ (2.86 Å), the plane (002) of ZnO (2.59 Å), and the plane (002) of CuO (2.52 Å) (Figure 1F). In addition, the rough surfaces of Zn_0.2_:Co_1_@Cu reveal abundant defects (Figure 1E), which are composed of missing link/cluster in crystal as well as residues of N for replacing O in metal oxide phases (Liu et al., 2019). The elemental mapping (Figure 1D) images illustrate that the Zn, Co, Cu, C, N, and O elements are uniformly distributed in the dodecahedral structure of Zn_0.2_:Co_1_@Cu, in which Cu is doped in dodecahedron due to the dissolution of a fraction of Cu foam. Thus multiple active sites would be composed of multicomponent metal and defects and activate CO_2_ molecules in reaction. Figures S1–S4 summarize the morphology and structures of heterojunctions Zn_x_:Co_y_@Cu, which confirm that various molar ratios of Zn to Co give rise to different crystal phases: Zn_0_:Co_1_@Cu to cubic Co_3_O_4_, Zn_1_:Co_1_@Cu to hexagonal ZnO, and cubic Co_3_O_4_, Zn_1_:Co_0_@Cu to hexagonal ZnO.

### PEC Performance of Photocathodes

**Figure 2:**
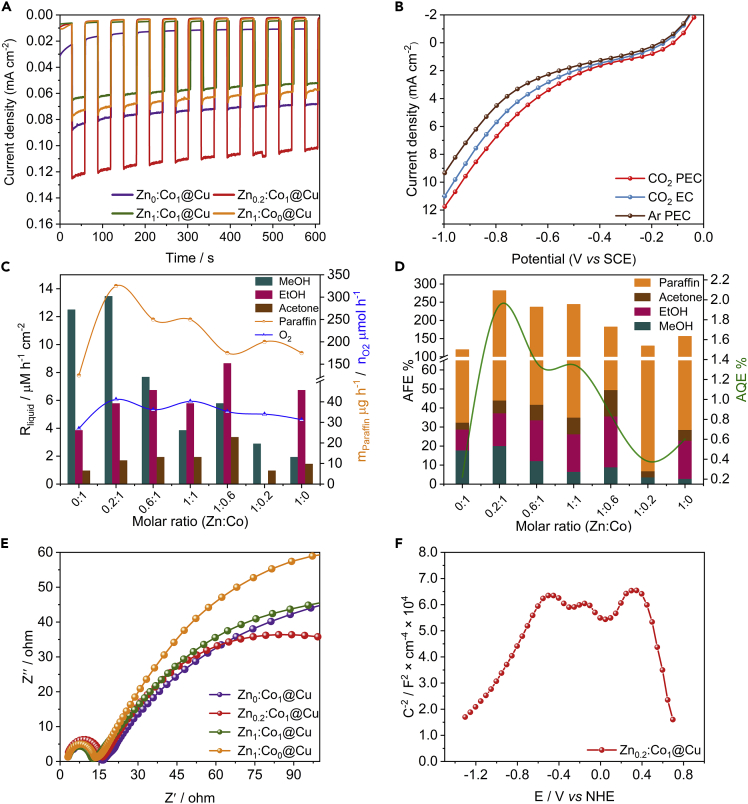
Catalytic Performances of Zn_x_:Co_y_@Cu in PEC Cell (A) Photocurrent densities as a function of Zn_x_:Co_y_@Cu at −1 V in the two-electrode system. (B) Linear sweep voltammetry curves of Zn_0.2_:Co_1_@Cu electrodes under EC and PEC conditions. See also Figure S8. (C) The evolution rate of hydrocarbon and O_2_ under the different molar ratio of Zn to Co photocathodes at −0.4 V versus SCE. See also Video S1. (D) Apparent faradaic efficiency (AFE) and apparent quantum efficiency (AQE) of PEC cells. (E) Nyquist plots of different photocathodes. (F) M-S plot of Zn_0.2_:Co_1_@Cu photocathode. See also Figure S9.

The photocurrent densities of representative four photocathodes show excellent characteristic curves of photoelectron response (Figure 2A), in which photocathode of Zn_0.2_:Co_1_@Cu gives the highest photocurrent. The linear sweep voltammetry curves (Figure 2B) show that the current density under PEC condition is much higher than that under argon and EC condition. Furthermore, the cyclic voltammetry curves demonstrate that the CO_2_ reduction peak of photocathode Zn_0.2_:Co_1_@Cu is approximately at −0.4 V versus saturated calomel electrode (SCE) (Figure S5).

We investigate the performance of CO_2_ reduction by using PEC cells of Zn_x_:Co_y_@Cu |KHCO_3_|BiVO_4_. Under potential of −0.4 V versus SCE, liquid products such as methanol (MeOH), ethanol (EtOH), and acetone can be detected in almost all PEC cells (Figure S13). The PEC cell of Zn_0.2_:Co_1_@Cu |KHCO_3_|BiVO_4_ gains the topmost yield of paraffin at a rate of 325 μg h^−1^ and releases O_2_ at a rate of 41 μmol h^−1^ (Figure 2C). The apparent quantum efficiency value of Zn_0.2_:Co_1_@Cu reaches 1.95%, which is about 9 times larger than that of the Zn_0_:Co_1_@Cu if its apparent faradaic efficiency equals 100% (Figure 2D). In addition, we test the activity of catalyst Zn_0.2_:Co_1_@Cu under different potentials from −0.2 to −1.3 V versus SCE (Figure S6). The paraffin products are generated under all potentials due to good C-C coupling control. Notably, this type of PEC system can tolerate voltages of up to −3.3 V (−1.0 versus SCE) to yield paraffin and release O_2_ at a rate of 181 μmol h^−1^ without H_2_ emission (Figure S7 and Video S1), which has possible potential application in industry.

Video S1. Oxygen Release in the Whole Online Reaction System, Related to Figure 2

To further understand the behavior of this effective PEC cell, its electrochemical impedance spectroscopy is obtained under EC condition (Figure 2E). The second arc radius of Zn_0.2_:Co_1_@Cu is smaller than others, which suggests a faster interfacial charge transfer from the cathode to electrolyte and favors the formation of active hydrogen atoms reducing CO_2_ to paraffin. Besides, Mott-Schottky (M-S) relationships of Zn_x_:Co_y_@Cu are gained to illustrate their semiconductor properties and carrier concentration (N_q_) (Figures 2F and S9). The slopes of the M-S curves present both positive and negative values for the Zn_x_:Co_y_@Cu cathode, implying the successful formation of p-n heterojunction of n-type ZnO and p-type Co_3_O_4_ on Cu foam (Cardon and Gomes, 1978). Furthermore, the N_q_ values of all cathodes can be calculated by the slopes of M-S curves, in which a lower slope of M-S curve reflects a higher N_q_ (Luo et al., 2013). Therefore, the N_q_ of the Zn_0.2_:Co_1_@Cu is higher than that of others (Figure S9D); this is the main reason why Zn_0.2_:Co_1_@Cu shows high photocurrent. Theoretically, the inner electric field will be built in the interfaces of p-n heterojunctions (Zhang et al., 2010). Specifically, charge carriers inside the Zn_x_:Co_y_@Cu are diffused and drifted between p-type Co_3_O_4_ (2.76 eV) and n-type ZnO (3.24 eV) (Mohamed Reda et al., 2017, Tak and Yong, 2008), thus forming a depletion layer at the interface. In this depletion layer a built-in electric field is formed due to the formation of positive and negative charges at the n- and p-sides, respectively. When the p-n heterojunction of Zn_x_:Co_y_@Cu is irradiated, it can enhance the concentration of carriers and tolerance for high external voltage, resulting in high efficiency of photogenerated electron-hole separation and excellent performance in CO_2_ reduction. UV-visible absorption spectra of Zn_x_:Co_y_@Cu catalysts reveal good absorption near UV to visible light (300–1,000 nm) (Figure S10). Therefore the heterojunctions not only ameliorate the ability of harvesting solar light but also obviously enhance the separation efficiency of photogenerated electron-holes.

### Paraffin Product Characterization

**Figure 3:**
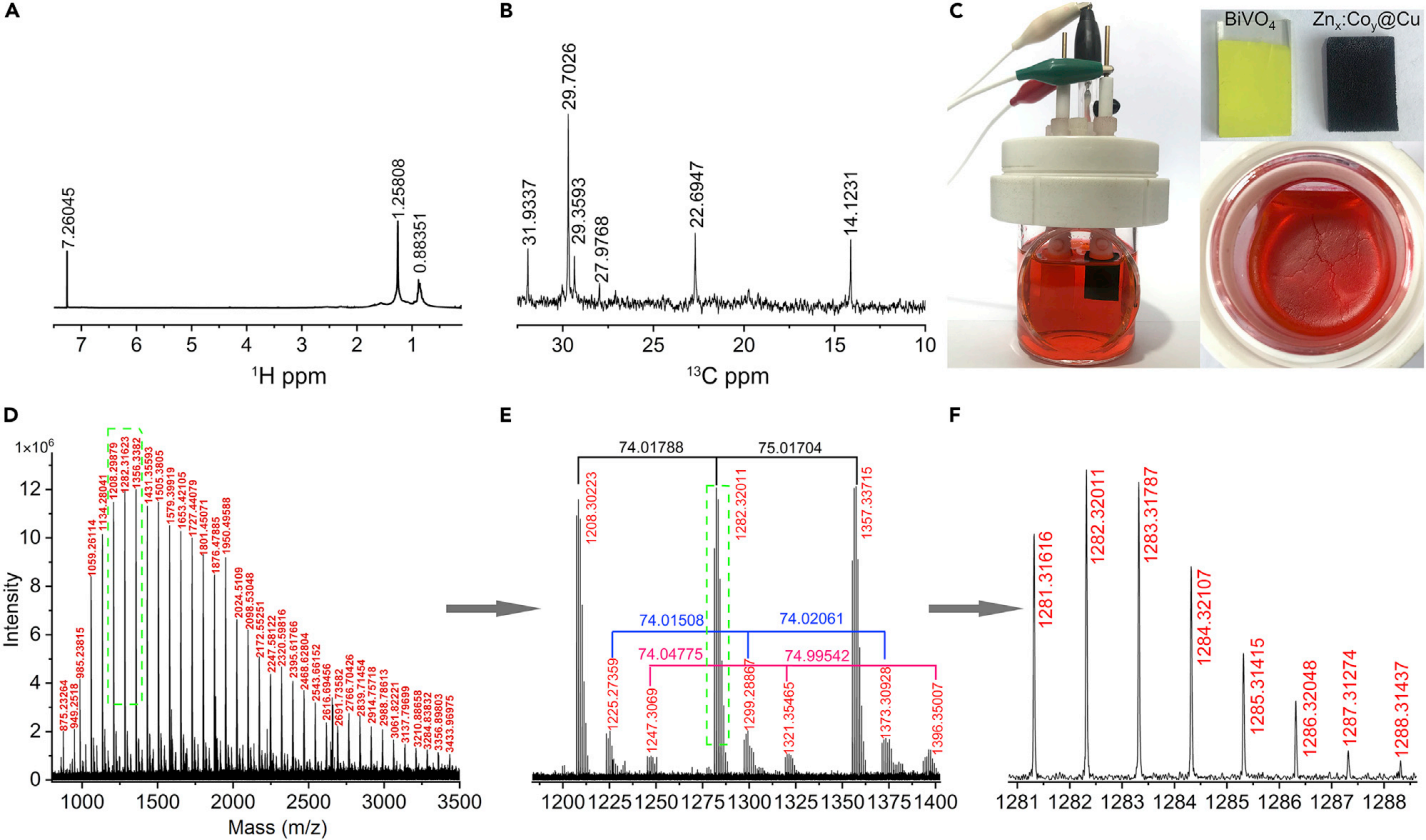
Paraffin Detection Paraffin is obtained through 4-h reaction under −0.4 V versus SCE using photocathode of Zn_0.2_:Co_1_@Cu. (A) ^1^H NMR spectrum of paraffin in CDCl_3_. (B) Corresponding ^13^C NMR. (C) PEC cell, photoelectrodes, and paraffin floated onto the electrolyte after reaction. See also Figure S12. (D) Complete MALDI-TOF MS spectra of ^13^C-paraffin. (E) Unit of C5 (74, 75 mass). (F) The isotopic distribution of ^13^C species. See also Figure S11.

To clarify the structure of paraffin product (Figure 3C), the ^1^H NMR, ^13^C NMR, and infrared spectra (Figures 3A, 3B, and S11B) are obtained. The typical peaks confirm a mixture of long-chain alkane compounds. The corresponding visible paraffin product is shown in Figure 3B; when the electrolyte was standing for several days, the transparent visible paraffin-like product floated on the surface of electrolyte (Figure S12). To trace the carbon source, ^13^CO_2_ labeling experiments are also carried out by using Zn_0.2_:Co_1_@Cu as photocathode. The distribution of molecular weight of the ^13^C labeling products is carefully examined by matrix-assisted laser desorption ionization time-of-flight mass spectrometry (MALDI-TOF MS). We can find that these paraffin products are a series of long-chain oxygen-containing hydrocarbons and their molecular weight increases from 875 to 3,434 (Figure 3D). As shown in Figure 3E, we speculate that these paraffin products might have the following structural formula: [^13^CH_2_OH(^13^CH_2_)_55_(CH_2_)_7_][(^13^CH_2_)_4_(CH_2_)]_3_CH_2_OH for 1208; [^13^CH_2_OH(^13^CH_2_)_54_(CH_2_)_7_][(^13^CH_2_)_4_(CH_2_)]_3_ [^13^CHOH] CH_2_OH for 1225, and [^13^CH_2_OH(^13^CH_2_)_54_(CH_2_)](^13^CH_2_)_20_ (CHOH)_2_ CH_2_OH for 1247. Therefore, we can see that the long chain grows step by step with a unit of C5 (74 (^13^CH_2_)_4_(CH_2_) or 75 (^13^CH_2_)_5_), which is like a unit of C6 glucose in the Calvin cycle of plant cell. Obviously, Figure 3F further illustrates that the peak with a molecular weight of 1,283 in the case of using ^13^CO_2_ as the carbon source is very strong; this means the PEC cell converts CO_2_ and H_2_O to paraffin products. The common experiment of CO_2_ reduction in PEC cell of Zn_0.2_:Co_1_@Cu|KHCO_3_|BiVO_4_ gives similar long-carbon-chain compounds: [CH_3_(CH_2_)_51_CH_2_OH][(CH_2_)_20_]_x_ (1039, 1320, 1601, 1882, x = 1, 2, 3, 4) and [CH_2_OH(CH_2_)_80_CH_2_OH] for 1183 (Figure S11A). These phenomena could be attributed to the proper 3D dodecahedra structure of heterojunction with multiple active sites favoring C-C coupling. The C_54_H_110_ (801) long-chain hydrocarbon is a basic unit that could be generated by the control of dodecahedral cage and multiple active sites for C-C coupling.

### Structure Analyses via X-Ray Absorption Fine Structure Spectroscopy about Zn_0.2_:Co_1_@Cu

**Figure 4:**
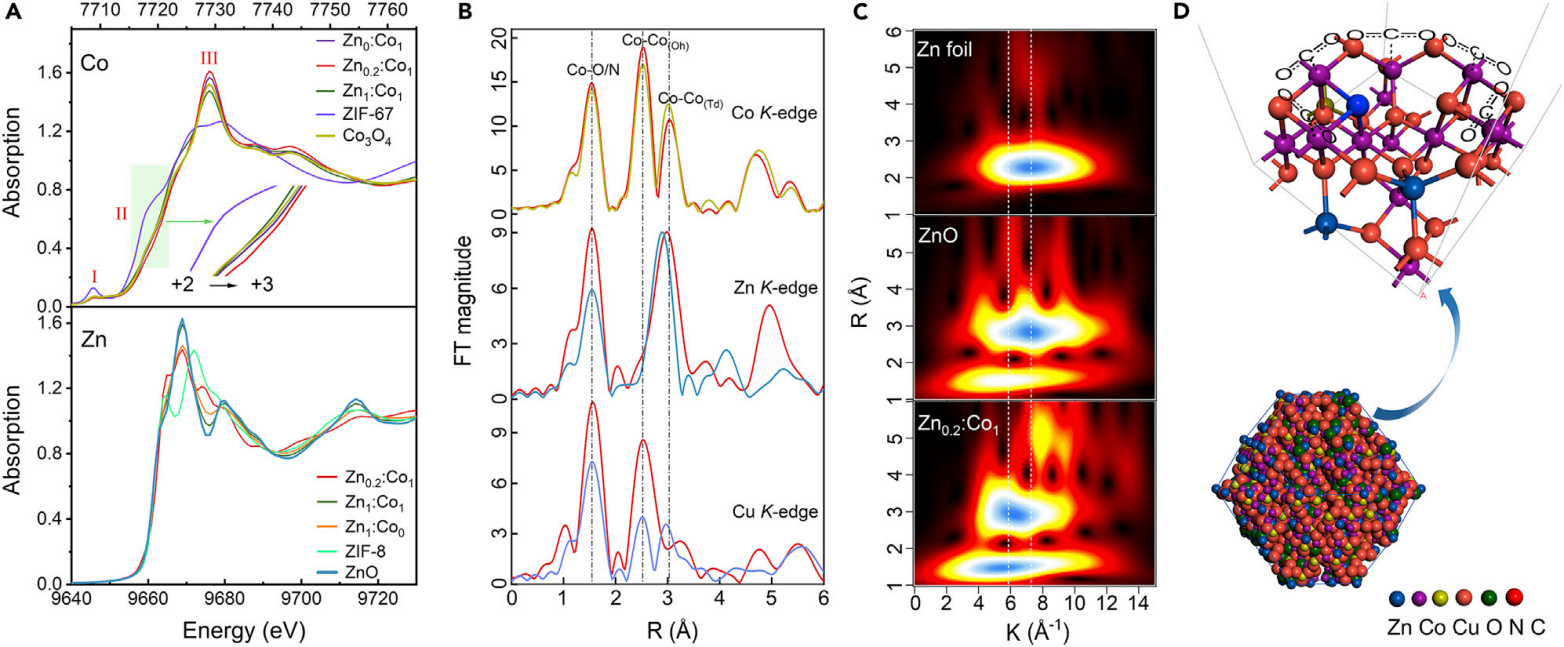
Results of XAFS Spectroscopy and Depicted Structures for Zn_0.2_:Co_1_@Cu (A) The XANES spectra of Co and Zn for Zn_x_:Co_y_@Cu with prepared ZIF-67/8 and Co_3_O_4_, ZnO as references, respectively. (B) The EXAFS spectra of Co, Zn, and Cu K-edge for Zn_0.2_:Co_1_@Cu with Co_3_O_4_, ZnO, and CuO as references, respectively. See also Figures S15–S18 and Tables S3–S5. (C) Wavelet transform spectra of Zn foil, ZnO, Zn_0.2_:Co_1_@Cu. (D) Crystal structure of Zn_0.2_:Co_1_@Cu dodecahedron and magnification of the corner unit that interacted with CO_2_.

For a deeper understanding of the relationship between the activity and the structural feature of catalysts, the fine valence states of Co, Zn, and Cu are revealed by X-ray absorption edge structure analyses. The Co K-edge X-ray absorption near-edge structure (XANES) spectra of Zn_x_:Co_y_@Cu exhibit similar features, indicating that Co_3_O_4_ phase is formed in each sample (Figure 4A). In detail, the illustration II in Figure 4A shows that the average valence state of Co elements in Zn_0.2_:Co_1_@Cu is higher than that in others. The octahedral coordination has a lower intensity at peak I than the tetrahedron one, as well as a higher intensity at peak III in Figure 4A. These imply that some Co^2+^ cations in the lattice of crystal Co_3_O_4_ are replaced by Zn^2+^ cations (Rong et al., 2015) (Figure S14). The Zn K-edge XANES spectrum of Zn_0.2_:Co_1_@Cu appears as an absorption edge energy at 9,668.9 eV that is typical characteristics of Zn^2+^. The two small shoulders at 9,664.8 and 9,674.4 eV indicate that some tetrahedra of Zn-N are successfully retained in this catalyst compared with the curve of ZIF-8. To further investigate the structure, Fourier transforms of Co, Zn, and Cu extended X-ray absorption fine structure (EXAFS) spectra of the Zn_0.2_:Co_1_@Cu catalyst are showed in Figure 4B. It is noted that the second peak of Zn K-edge has a slight shift to 3 Å and a new peak appears around 5 Å compared with ZnO, which suggests a structural distort compared with other catalysts. The Cu K-edge gives rise to a very intense peak at about 2.5 Å, which is similar to the observed peak in the Co K-edge; this means that amounts of Co^2+^ are substituted by Cu^2+^ in the Co_3_O_4_ structure. As strong evidences, the wavelet transform plots (Figure 4C) show the maximum intensity related to Zn-Zn bonding at ∼7 Å^−1^ in Zn foil and ZnO. The signal of Zn-Zn in Zn_0.2_:Co_1_@Cu shifts to ∼6 Å^−1^, which explains the formation of Zn-Co bond. Besides, the new weak signal at ∼8 Å^−1^ is related to the peak of 5 Å in the Zn EXAFS spectrum; it could be attributed to the nanostructure commutative replacement in the lattices of ZnO and Co_3_O_4_ phases, which is well consistent with the HRTEM analysis. Hence, it is concluded that some Co^2+^ cations in the tetrahedral units of spinal Co_3_O_4_ have been successfully replaced by Zn^2+^ and Cu^2+^ in Zn_0.2_:Co_1_@Cu heterojunction. A dodecahedron structure related to the above results and elemental mapping images are illustrated in Figure 4D. In the (100) plane of Zn_0.2_:Co_1_@Cu, five CO_2_ molecules could be captured by Co atoms and connected each other in the CO_2_ reduction process as well as in plant cell, where the Zn^2+^ and Cu^2+^ dispersed well in samples resulting in a cooperative effect.

### Proposed Mechanism of CO_2_ Reduction at Zn_x_:Co_y_@Cu |KHCO_3_|BiVO_4_

**Figure 5:**
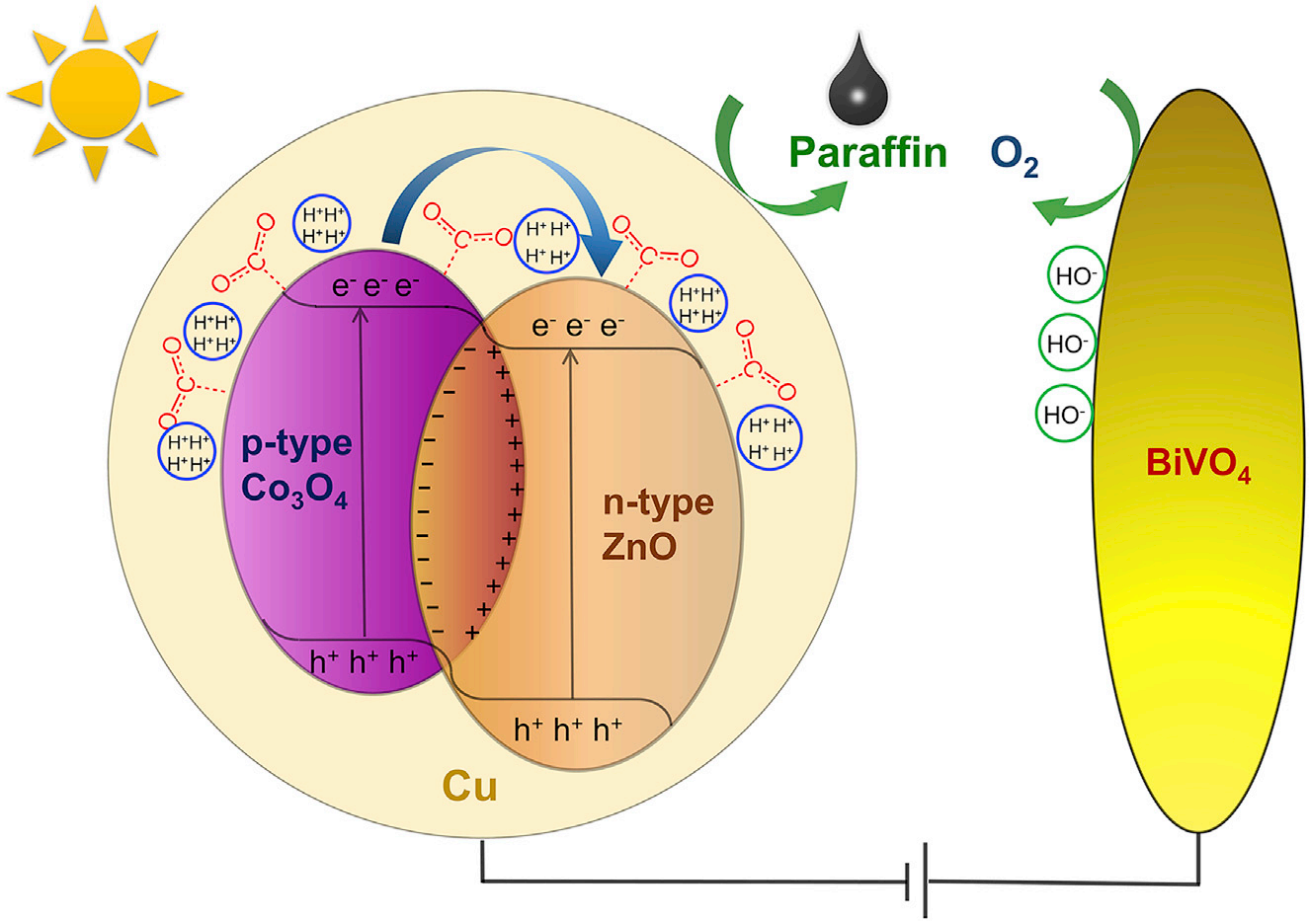
A Proposed Mechanism for the Artificial Photosynthesis of Paraffin

According to the experimental results and characterizations, a possible mechanism for the PEC reduction of CO_2_ to paraffin product is proposed and illustrated in Figure 5. Protons could move to the Zn_x_:Co_y_@Cu photocathode under a low-bias potential and converted to active hydrogen atoms by high-energy photoelectrons in semiconductors. On the other hand, hydroxyl groups (OH^−^) transfer to the photoanode of BiVO_4_ releasing O_2_ (Seabold and Choi, 2012). When light irradiation is applied to this PEC cell, photogenerated electrons (e^−^) and holes (h^+^) can be generated in Zn_x_:Co_y_@Cu and quickly separated by the built-in electric field resulting in higher mobilities of charge carriers. Simultaneously, the electrons could be transferred from the conduction band (CB) of Co_3_O_4_ to the CB of the n-type ZnO and the holes are captured by electrons from the circuit or OH^−^. Hence, the high concentration of photoelectrons was captured by protons in the surfaces to form abundant active hydrogen atoms that could reduce multiple CO_2_ molecules into paraffin as the Calvin cycle in plant. In this process, the H_2_ release is suppressed due to the rapid rate of CO_2_ reduction at multiple active sites of metals and nitrogen.

### Conclusion

In summary, for the first time we demonstrate a strategy to construct C/N-doped Zn_x_:Co_y_@Cu heterojunctions as photocathodes, which mimic the Calvin cycle of plants and achieve multiple C-C couplings in our PEC cell to generate the paraffin product. At the optimal photocathode of Zn_0.2_:Co_1_@Cu, some Co^2+^ cations in the tetrahedral units of spinal Co_3_O_4_ have been successfully replaced by Zn^2+^ and Cu^2+^ and form favorable heterojunctions, in which CO_2_ molecules are adsorbed and activated by multiple active sites of metals and nitrogen and reduced to paraffin product by active hydrogen atoms. Although there are still many challenges in applying our system to industrial paraffin product, an understanding of the mechanism could provide different perspectives for CO_2_ reduction.

### Limitations of the Study

• It is very difficult to observe and analyze a specific catalytic reaction with multiple active sites in water.
• The catalytic mechanism of artificial photosynthesis for paraffin could not be verified by theoretical calculations due to the complexity of the catalytic system and multiple coupling of carbon-based species.

## Methods

All methods can be found in the accompanying Transparent Methods supplemental file.

## Supplemental Information

Document S1. Transparent Methods, Figures S1–S18, and Tables S1–S5
