# Supplementary material for: Photoelectrocatalytic Reduction of CO2 to Paraffin Using p-n Heterojunctions
Source: iScience. 2019 Dec 12;23(1):100768. doi: 10.1016/j.isci.2019.100768 (PMC6941872; doi:10.1016/j.isci.2019.100768)
Supplement: Document S1. Transparent Methods, Figures S1–S18, and Tables S1–S5 [file mmc1.pdf]

ISCI, Volume *23*

Supplemental Information

Photoelectrocatalytic Reduction of CO_2 to Paraffin Using p-n Heterojunctions

Jinyuan Wang, Yongji Guan, Xiaogang Yu, Youzhi Cao, Jiazang Chen, Yilin Wang, Bin Hu, and Huanwang Jing

Supplemental Information

Supplemental Figures and Tables

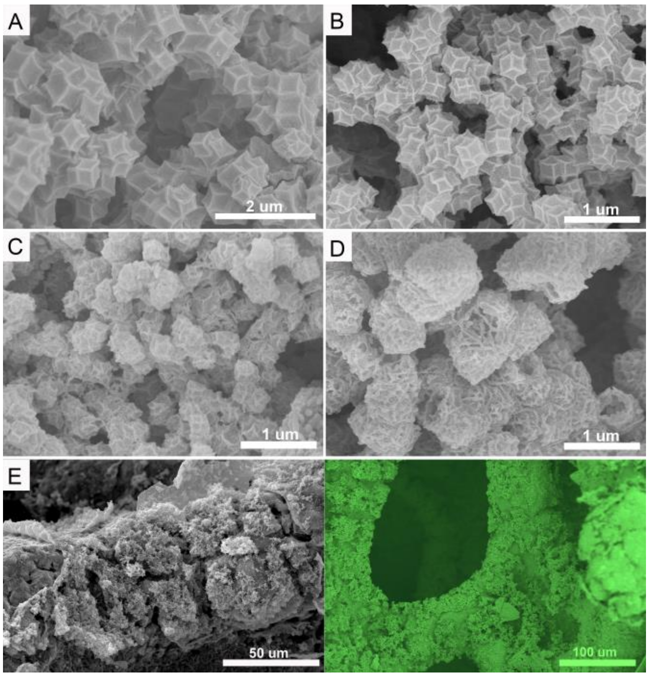

Figure S1. SEM images of Zn_x:Co_y@Cu. A, Zn_0:Co_1@Cu, **B,** $Zn_{0.2}$:Co_1@Cu, **C,** Zn_1:Co_1@Cu, **D,** Zn_1:Co_0@Cu and **E,** large thumbnails of $Zn_{0.2}$:Co_1@Cu. Related to Figure 1.

The SEM image of the Zn_x:Co_y@Cu suggested that the diameters of Zn_0:Co_1@Cu and Zn_1:Co_0@Cu are nearly 1 μm. The volume of bimetallic Zn_x:Co_y@Cu is relatively small, the diameter of $Zn_{0.2}$:Co_1@Cu and Zn_1:Co_1@Cu are about 500 nm. As the proportion of Zn in the bimetallic Zn_x:Co_y@Cu increases, it changes from a regular polyhedron to a surface-porous irregular particle. The zeolitic imidazolate frameworks (ZIFs) is uniformly loaded on the Cu foam.

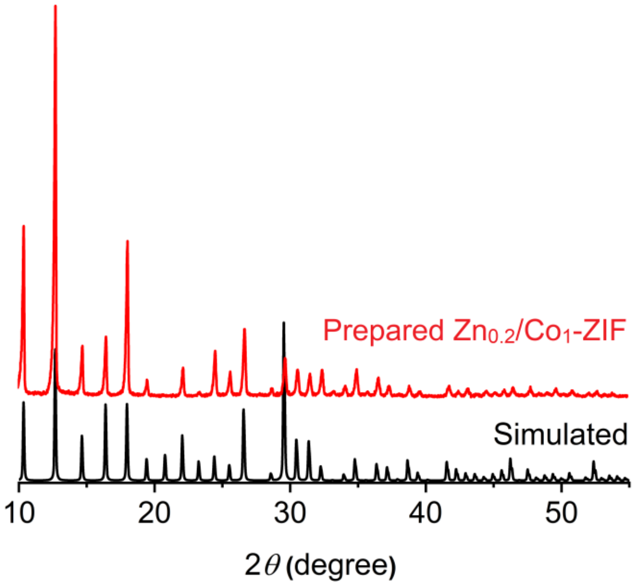

Figure S2. The XRD patterns of as-prepared $Zn_{0.2}$/Co_1-ZIFs sample. Related to Figure 1.

The prepared XRD pattern matches well with the simulated one which is corresponding to a sodalite structure of pure cubic ZIF-8 as literature reported(Fairen-Jimenez et al., 2011; Qin et al., 2017). The sharp and strong characteristic peaks reflect the high crystallinity of the material prepared with the synthesizing protocol.

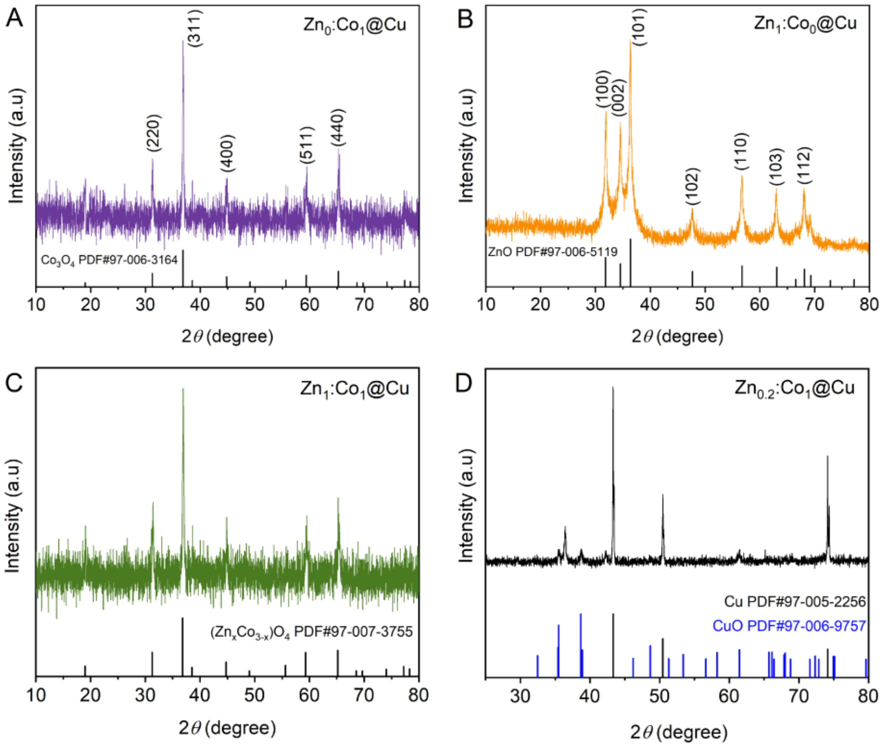

Figure S3. XRD patterns of **A**, Zn_0:Co_1, **B**, Zn_1:Co_0, **C**, Zn_1:Co_1 and **D** $Zn_{0.2}$:Co_1@Cu. Related to Figure 1.

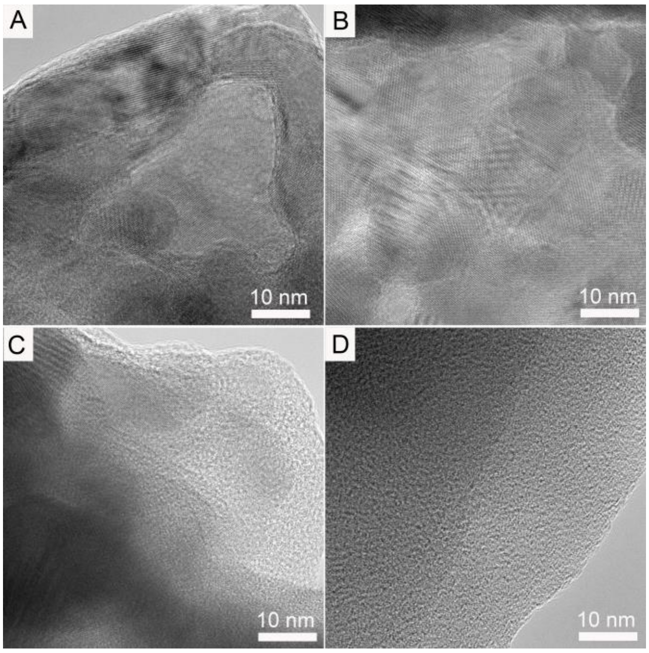

Figure S4. HRTEM images of **A**, Zn_0:Co_1@Cu, **B**, $Zn_{0.2}$:Co_1@Cu, **C**, Zn_1:Co_1@Cu and **D** Zn_1:Co_0@Cu. Related to Figure 1.

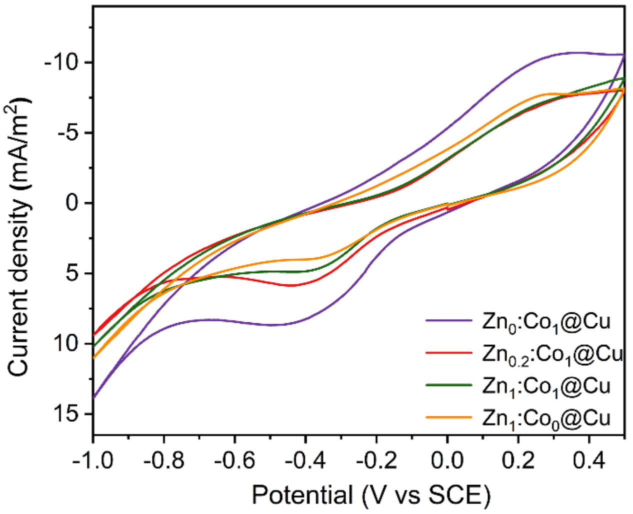

Figure S5. CV curves of Zn_x:Co_y@Cu in PEC condition. Related to Figure 2 and Figure S6.

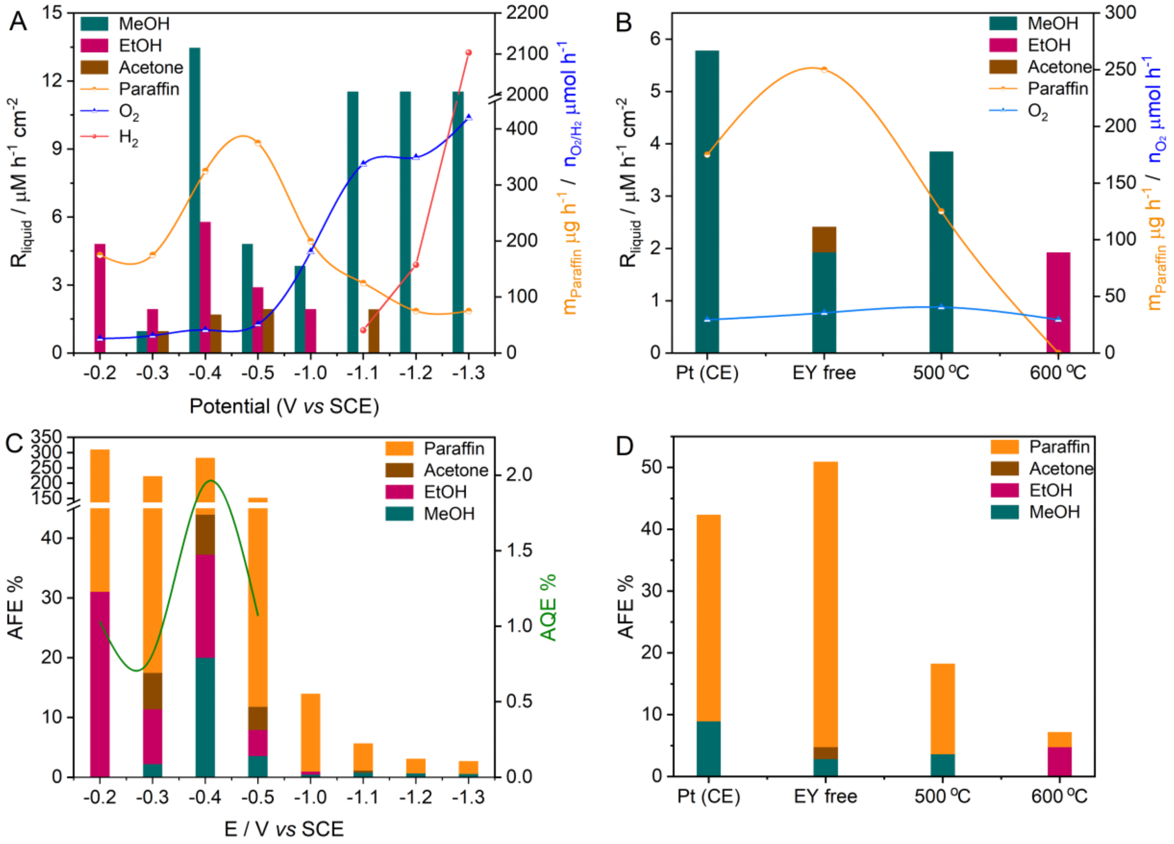

Figure S6. Comparison of catalytic performance. A, C, rate and efficiency (AQE, AFE) of the PEC activities of $Zn_{0.2}$:Co_1@Cu at different potential. **B, D,** rate and efficiency under the follow condition: Pt foil as a photoanode; the electrolyte do not contain EY; as well as pyrolysis $Zn_{0.2}$/Co_1-ZIFs under 500ºC and 600ºC, respectively. The AQE is not calculated in the case of the AFE is less than 100%. Related to Figure 2 and Figure S5.

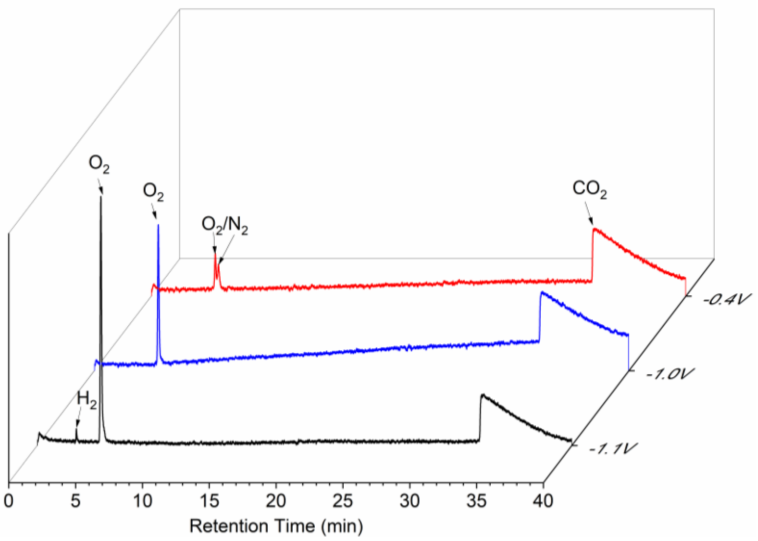

Figure S7. Representative GC data showing the signal of H_2 appeared under -1.1V *vs.* SCE. Related to Figure 2 and Figure S6.

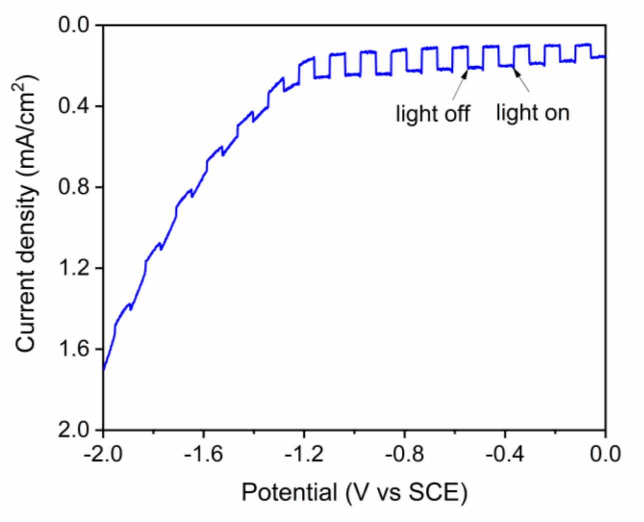

Figure S8. Photocurrent densities by solar irradiation of $Zn_{0.2}$:Co_1@Cu | $KHCO_3$| $BiVO_4$ under the PEC condition with chopped light. Related to Figure 2.

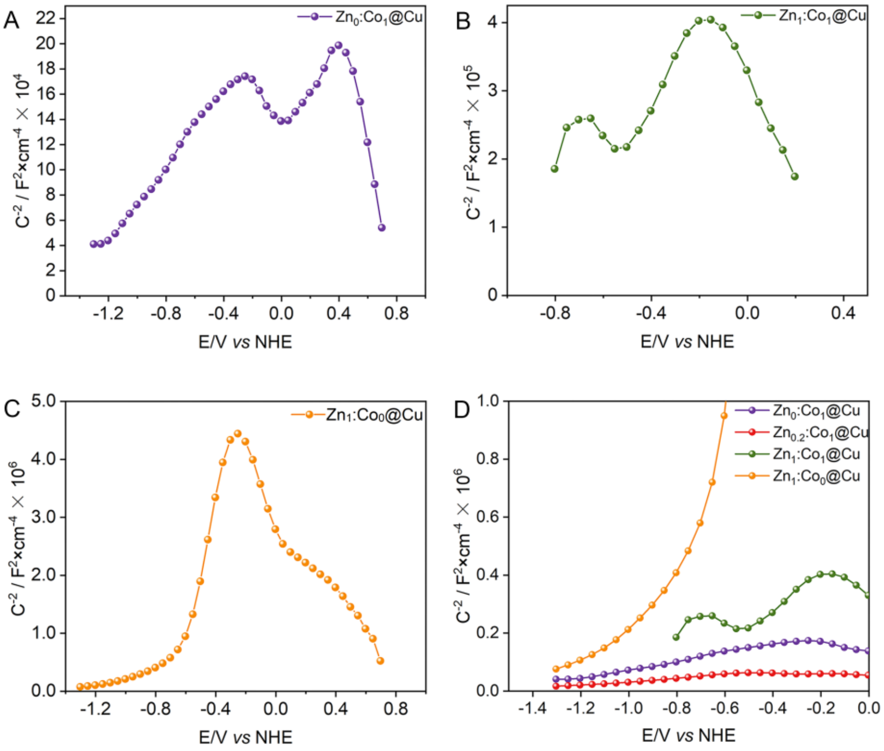

Figure S9. M-S plots of Zn_x:Co_y@Cu electrode. M–S analysis is carried out with Zn_x:Co_y@Cu work electrodes, Pt foil counter electrode and reference electrode Ag/AgCl (Sat. KCl). The electrolyte contains 0.1 M $KHCO_3$ solution and non-CO_2 bubble. After that, M-S plots are generated based on capacitance that is derived from the electrochemical impedance obtained at each potential with the frequency of 0.5 kHz under dark. Related to Figure 2.

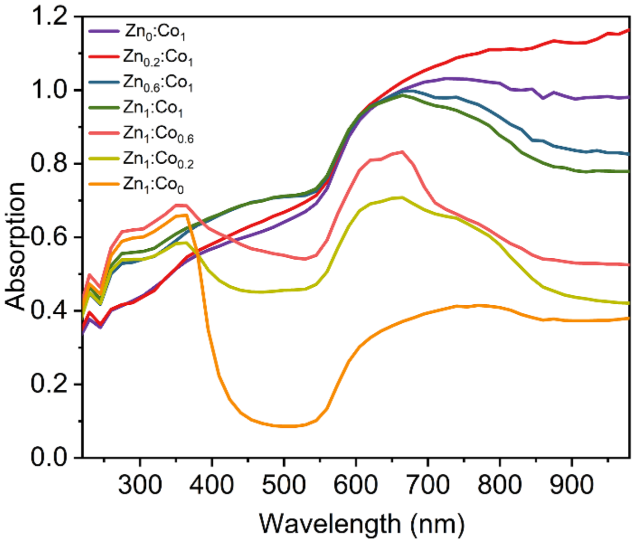

Figure S10. The solid-state UV-vis absorption spectra of the photocathodes Zn_x:Co_y@Cu. Related to Figure 2.

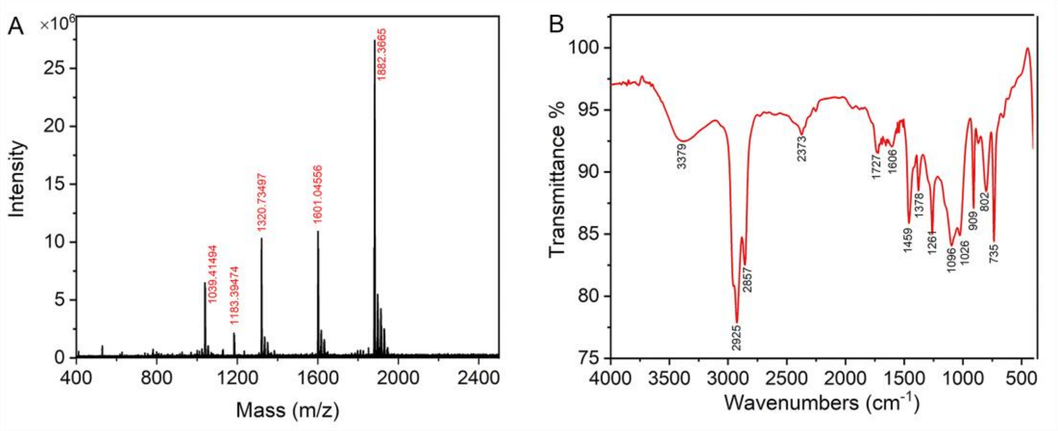

Figure S11. Comparison for paraffin products using $^{12}CO_2$ as raw materials. A, MALDI-TOF MS analysis about paraffin which is obtained after reaction of 4h under -0.4V versus SCE using $Zn_{0.2}$:Co_1@Cu as photocathode. **B**, the corresponding infrared (IR) spectroscopy analysis. Related to Figure 3 and Figure S12.

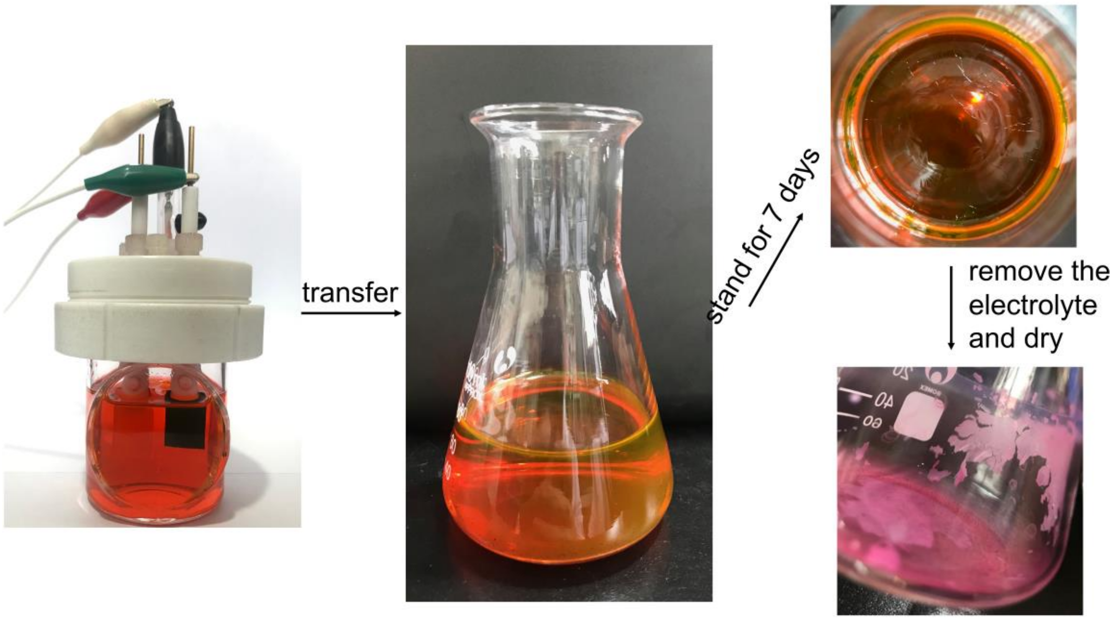

Figure S12. Paraffin product photos. Related to Figure 3 and Figure S11.

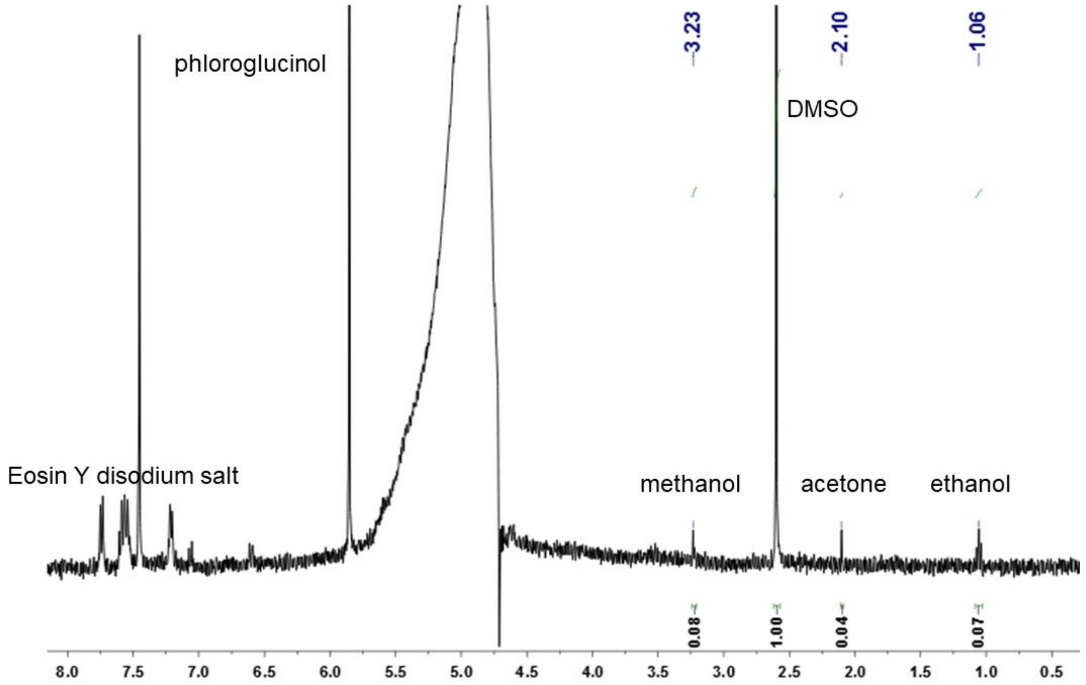

Figure S13. Representative NMR spectrum showing the signals of the liquid product of PEC CO_2 reduction: methanol, ethanol, acetone, Eosin Y disodium salt, and the internal standard of DMSO and phloroglucinol. The concentration of methanol, ethanol and acetone was calculated based on the ratio of the integrals of formate to DMSO. Related to Figure 2, Figure S6 and Figure S7.

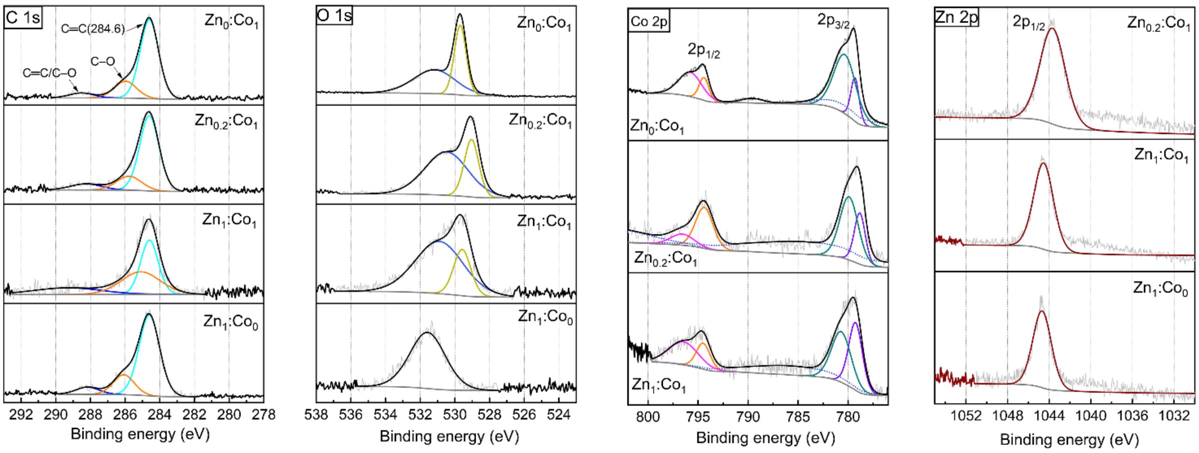

Figure S14. X-ray photoelectron spectroscopy of Zn_x:Co_y@Cu. The chemical bonding energy of C 1s, O 1s, Co 2p and Zn 2p for Zn_x:Co_y@Cu. Compared to Zn_0:Co_1@Cu, a negative shifts is observed in $Zn_{0.2}$:Co_1@Cu sample for Co $2p_{3/2}$. Similarly, a negative shift is observed in $Zn_{0.2}$:Co_1@Cu for Zn $2p_{1/2}$ compared with Zn_1:Co_0@Cu. The explanation of the shift is probably due to the incorporation of Zn^{2+} into Co_3O_4. Related to Figure 4.

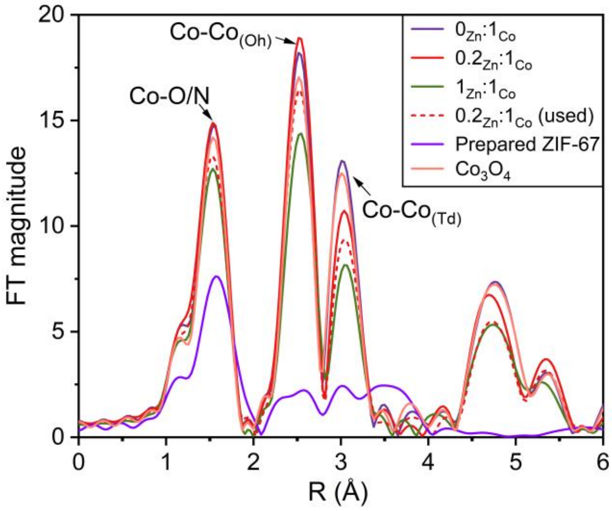

Figure S15. The EXAFS spectra of Co K-edge for Zn_x:Co_y@Cu, Co_3O_4 and prepared ZIF-67, respectively. Related to Figure 4.

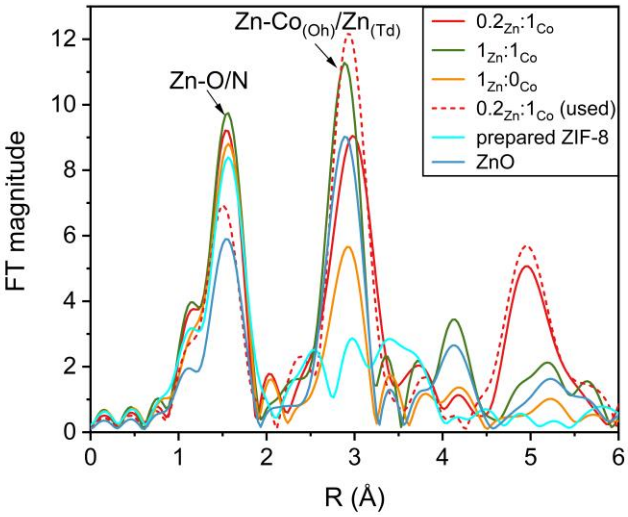

Figure S16. The EXAFS spectra of Zn K-edge for Zn_x:Co_y@Cu, ZnO and prepared ZIF-8, respectively. Related to Figure 4.

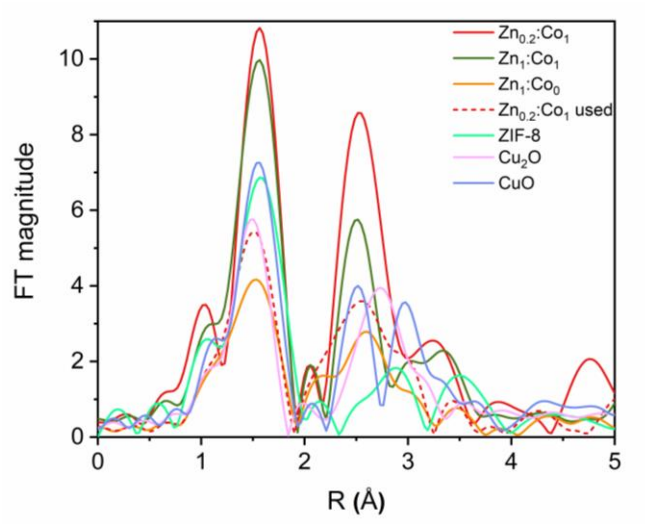

Figure S17. The EXAFS spectra of Cu K-edge for Zn_x:Co_y@Cu, CuO and CuO_2, respectively. Related to Figure 4.

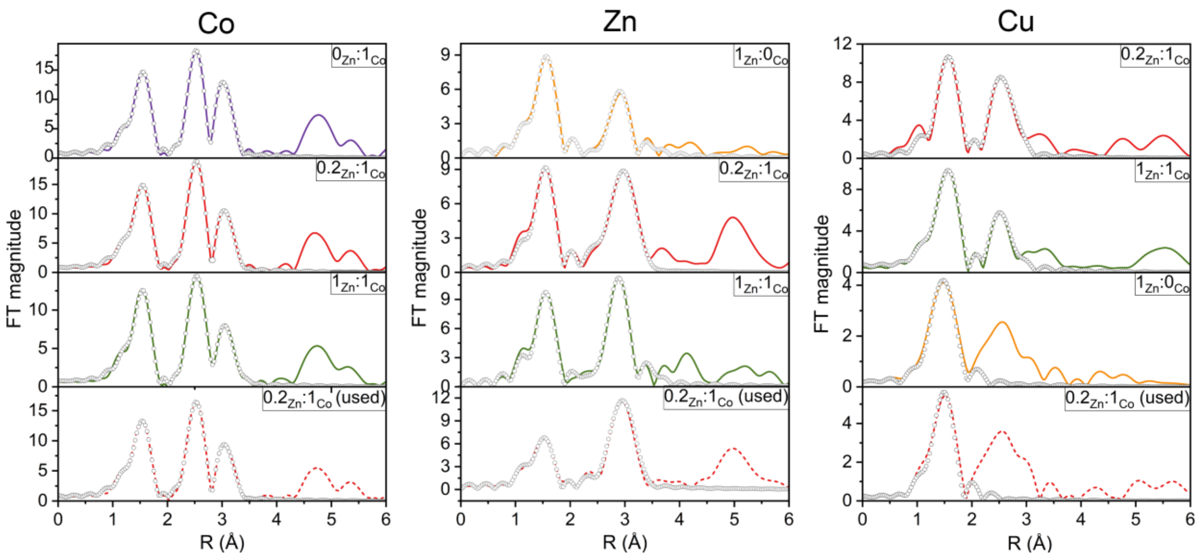

Figure S18. EXAFS fitting curve for Zn_x:Co_1@Cu. Related to Figure4.

Table S1. Elemental analysis using ICP-OES for Co, Zn and Cu with different samples (mass%). The data are average value of three times. Related to Figure 1.

Sample	Co(%)	Zn(%)	Cu(%)
Zn_0:Co_1	78.47	0	1.442
$Zn_{0.2}$:Co_1	48.61	17.79	10.295
Zn_1:Co_1	24.07	55.80	1.709
Zn_1:Co_0	0	65.65	0.753

Table S2. BET surface areas and pore volumes of the samples. BET surface area calculations were based on the adsorption isotherms using P/P_0 from 0.005 to 0.1. Related to Figure 1.

Sample	S_{BET} (m^2/g)[a]	V_{tatal} (cm^3/g)	V_{mic} (cm^3/g)	Aperture
$Zn_0:Co_1$	7.5060	0.0376	0.0022	31.7002
$Zn_{0.2}:Co_1$	15.4993	0.0819	0.0016	28.1005
$Zn_1:Co_1$	7.8388	0.0280	0.0024	36.6862
$Zn_1:Co_0$	12.5728	0.0279	0.0030	19.1169

Table S3. EXAFS fitting parameters at the Co K-edge for various sample. [a] *N*: correction. *R* factor: goodness of fit. S_0^2, 0.77, was obtained from the experimental EXAFS fit of Co_3O_4 reference by fixing CN as the known crystallographic value and was fixed to all the samples. Related to Figure 4.

Sample	Shell	*N* [a]	*R* (Å) [b]	σ^2 ($Å^2 \cdot 10^3$) [c]	ΔE^0 (eV) [d]	*R* factor (%)
$Zn_0:Co_1$	Co-O	5.6	1.92	3.2	5.4	0.2
	Co-Co	4.4	2.86	3.3	4.4	
	Co-Co	10.4	3.37	7.1	1.9	
$Zn_{0.2}:Co_1$	Co-O	6.1	1.92	3.6	5.1	0.2
	Co-Co	5.5	2.86	4.1	3.6	
	Co-Co	7.7	3.37	6.5	3.9	
$Zn_1:Co_1$	Co-O	5.5	1.92	4.0	4.4	0.3
	Co-Co	3.8	2.86	3.8	3.4	
	Co-Co	9.7	3.37	10.4	6.4	
$Zn_{0.2}:Co_1$ (used)	Co-O	5.5	1.91	3.6	4.4	0.3
	Co-Co	4.9	2.86	4.2	3.6	
	Co-Co	6.8	3.37	6.5	3.6	

Table S4. EXAFS fitting parameters at the Cu K-edge for various samples. [a] *N*: coordination numbers; [b] *R*: bond distance; [c] σ^2: Debye-Waller factors; [d] ΔE_0: the inner potential correction. *R* factor: goodness of fit. S_0^2, 0.75, was obtained from the experimental EXAFS fit of CuO reference by fixing CN as the known crystallographic value and was fixed to all the samples. Related to Figure 4.

Sample	Shell	*N* [a]	*R* (Å) [b]	σ^2 (Å^2 ·10^3) [c]	ΔE^0 (eV) [d]	*R* factor (%)
Zn_1:Co_0	Cu-O	2.1	1.89	6.1	8.9	0.7
$Zn_{0.2}$:Co_1	Cu-O	4.1	1.95	2.1	6.4	2.0
	Cu-Cu	4.7	2.89	6.5	3.2	
Zn_1:Co_1	Cu-O	5.4	1.96	5.7	7.3	1.0
	Cu-Cu	2.2	2.86	4.5	-2.0	
$Zn_{0.2}$:Co_1 (used)	Cu-O	2.4	1.89	4.8	7.5	1.6
CuO	Cu-O	**4**	1.94	4.9	7.2	0.3

Table S5. EXAFS fitting parameters at the Zn K-edge for various samples. [a] *N*: coordination numbers; [b] *R*: bond distance; [c] σ^2: Debye-Waller factors; [d] ΔE_0: the inner potential correction. *R* factor: goodness of fit. S_0^2, 0.77, was obtained from the experimental EXAFS fit of ZnO reference by fixing CN as the known crystallographic value and was fixed to all the samples. Related to Figure 4.

Sample	Shell	*N* [a]	*R* (Å) [b]	σ^2 (Å^2 ·10^3) [c]	ΔE0 (eV) [d]	*R* factor (%)
Zn_1:Co_0	Zn-O	0.9	1.86	1.3	3.9	0.8
	Zn-O	2.7	1.98	1.0		
	Zn-Zn	3.4	3.23	8.0	4.9	
$Zn_{0.2}$:Co_1						2.0
	Zn-O	3.7	1.96	3.5	3.7	
	Zn-Zn	19.2	3.33	17.0	-4.7	
Zn_1:Co_1	Zn-O					1.1
	Zn-O	4.0	1.97	3.9	4.6	
	Zn-Zn	10.6	3.22	11.0	2.5	
$Zn_{0.2}$:Co_1 (used)	Zn-O					4.0
	Zn-O	4.5	1.96	8.2	-0.5	
	Zn-Zn	10.0	3.32	9.0	-8.2	
ZnO	Zn-O	**1**	1.88	0.2	5.5	0.5
	Zn-O	**3**	1.99	0.9		
	Zn-Zn	**12**	3.22	9.6	2.9	

The acquired EXAFS data were processed according to the standard procedures using the ATHENA module implemented in the IFEFFIT software packages. The k^3-weighted EXAFS spectra were obtained by subtracting the post-edge background from the overall absorption and then normalizing with respect to the edge-jump step. Subsequently, k^3-weighted $\chi(k)$ data of K-edge were Fourier transformed to real (R) space using a hanning windows (dk=1.0 Å^{-1}) to separate the EXAFS contributions from different coordination shells. To obtain the quantitative structural parameters around central atoms, least-squares curve parameter fitting was performed using the ARTEMIS module of IFEFFIT software packages.

Transparent Methods

Chemicals and materials.

Carbon dioxide (CO_2) gas was purchased as 99.999% purity. $^{13}CO_2$ was obtained from Aldrich Company. Tetrabromofluorescein, DMSO, phloroglucinol, Eosin Y disodium salt, potassium bicarbonate, 2-Methylimidazole, Zinc (II) nitrate hexahydrate ($Zn(NO_3)_2{\cdot}6H_2O$) and Cobalt(II) nitrate hexahydrate ($Co(NO_3)_2{\cdot}6H_2O$) were ordered from Sinopharm Chemical Reagent Co., Ltd. D_2O were purchased from Cambridge Isotope Laboratories, Inc. Other chemicals and reagents were purchased from Adamas-beta® as analytical grade commercial products and used for reaction without further purification unless otherwise indication.

Characterization.

X-ray diffraction (XRD) patterns of materials were recorded on an X'Pert PRO diffractometer. Scanning electron microscope (SEM) was performed on Apreo S. Transmission electron microscopy (TEM) and corresponding energy disperse spectroscopy (EDS) mapping were performed on FEI Talos F200s under accelerating voltage of 200 KV. X-ray photoelectron spectroscopy (XPS) of samples were measured using a VG Scientific ESCALB210-XPS photoelectron spectrometer equipped with an Mg Kα X-ray resource. For the core-level spectra, the carbon 1s feature located at 284.6.0 eV was used to calibrate the binding energies. Elemental analysis of metal quantity was detected by ICP-OES (PQ 9000). The Fourier transform infrared spectroscopy (FTIR) were recorded with KBr pellets in the range of 4000-400 cm^{-1} on a NEXUS 670 spectrometer. The optical absorption properties of electrodes were characterized by a UV–vis spectrophotometer (UV-2600, Shimadzu).

^1H and ^{13}C NMR spectra were recorded on a Varian AM-400 spectrometer using D_2O, DMSO and phloroglucinol as an internal standard. Gas chromatograph (GC) analyses of gaseous product were carried out on a Varian CP-3800 GC equipped with flame ionization detector (FID) and thermal conductivity detector (TCD) detectors. A solar simulator PLS-SXE300C was used as light source. A CHI660E electrochemical workstation was used for different parameters of experiments. A standard Si-solar cell of Fraunhofer ISE was used to calibrate the light density of solar simulator.

Preparation of Zn_x:Co_y-ZIF@Cu.

A series of samples are prepared with different Zn to Co molar ratios, which grow in situ on Cu foam and record as Zn_x/Co_y-ZIF@Cu, where x/y is the molar ratio of $Zn(NO_3)_2{\cdot}6H_2O$ to $Co(NO_3)_2{\cdot}6H_2O$ and their total moles are 3 mmol. All the photocathodes of Zn_x/Co_y-ZIF@Cu are prepared in the same method, and the preparation of $Zn_{0.2}$/Co_1-ZIFs@Cu is described in detail as following. The Cu foam is cut into a square electrode (1.5 × 2 cm), then is successively washed with isopropanol, ethanol, acetone, hydrochloric acid and deionized H_2O under ultrasonication for 30 min and natural drying before use. In a typical synthesis process, $Co(NO_3)_2{\cdot}6H_2O$ (148.5 mg, 0.5 mmol) and $Zn(NO_3)_2{\cdot}6H_2O$ (727.5 mg, 2.5 mmol) are dissolved in 30 ml methanol (MeOH) to form a clear solution. Then the Cu foam is soaked in above solution and 10 mL MeOH containing 2-methylimidazole (984 mmg, 12 mmol) is subsequently droped in 10 minutes. The reaction is incubated at room temperature for 24 h with slow stirring. The synthesized $Zn_{0.2}$/Co_1-ZIF@Cu is washed with ethanol for several times and goes through natural drying overnight. The other mole ratio of Zn_x/Co_y-ZIF@Cu have prepared with the same process as mentioned above.

Preparation of Zn_x:Co_y@Cu.

The Zn_x:Co_y@Cu are placed in a tube furnace and then heat up to 400 ^oC for 30 min with a ramp of 5 ^oC min^{-1} under argon gas flow. After that, the argon gas is switched off, and the furnace is still kept in air at this temperature for another 30 min. Thus, the photocathode materials of Zn_x:Co_y@Cu are prepared.

Photoelectrochemical measurement.

Photoelectrochemical measurements are carried out with a CHI660E electrochemical workstation in actual three-

electrode PEC reaction condition in order to detect the state of the real reaction. The electrochemical impedance spectroscopy (EIS) experiments are conducted with amplitude of 10 mV and frequency ranging from 0.001 Hz to 1 MHz in 0.1 M $KHCO_3$ solution (CO_2 saturation). E/V *vs*. RHE = E (*vs*. Hg/$HgCl_2$) + 0.242V +0.0591 × pH (6.8).

PEC activity measurement.

The PEC experiments are carried out in a PEC cell of Zn_x:Co_y@Cu| $KHCO_3$| $BiVO_4$ using SCE as reference electrode. The above PEC cell contains $KHCO_3$ aqueous solution (0.1 M; 80 mL) with or without Eosin Y disodium salt (0.01 M) as sensitizer and then the solution is saturated with a stream of high purity CO_2 for 30 min (pH = 6.8). After that, the PEC experiment is conducted under irradiation of simulated sunlight (AM 1.5G (Perfectlight), 200 mW cm^{-2}) with different potential. The liquid products are quantified by NMR spectroscopy and the ^{1}H spectrum are measured with H_2O suppression using a pre-saturation method, in which appropriate electrolyte is mixed with 35 μL standard liquid (1 mL D_2O + 7 mM dimethyl sulfoxide + 36 mM phloroglucinol). The gas products are analyzed using gas chromatograph (GC). The paraffin products are detected with MALDI-TOF MS.

Rate and apparent quantum efficiency (AQE) measurement.

The rate and efficiency (AQE and AFE) are measured under the ordinary PEC reaction condition and calculated according to the following equation,

$$\text{Rate (liquid product)} = \frac{C_{DMSO}(\mu M) \times \text{Relative Area}_{product}}{\text{Time(h)} \times 1.82(cm^{-2})}$$

$$\text{AFE (\%)} = \frac{\Sigma\ (\text{moles product} \times \text{electron transfer number(n)})}{\text{moles of electrons counted}}$$

$$\text{AQE (\%)} = \frac{\Sigma\ (\text{moles product} \times n)\ -\text{moles of electrons counted}}{\text{moles of photons irradiated on electrode}}$$

The moles of photons irradiated on electrode

$$= \frac{\text{light density(mW cm}^{-2}) \times \text{suface area(cm}^2) \times \text{radiation time(s)}}{\text{photon (500 nm) energy(J)} \times 6.02 \times 10^{23}}$$

XAFS measurements.

The X-ray absorption fine structure spectra (XAFS) are collected at 1W1B station in Beijing Synchrotron Radiation Facility (BSRF). The storage rings of BSRF is operated at 2.5 GeV with a maximum current of 250 mA. Using Si (111) double-crystal monochromator, the data collection is carried out in transmission mode using ionization chamber. All spectra are collected in ambient condition.

Supplementary References

Fairen-Jimenez, D., Moggach, S.A., Wharmby, M.T., Wright, P.A., Parsons, S., and Düren, T. (2011). Opening the Gate: Framework Flexibility in ZIF-8 Explored by Experiments and Simulations. J Am Chem Soc *133*, 8900-8902.

Qin, J., Wang, S., and Wang, X. (2017). Visible-light reduction CO_2 with dodecahedral zeolitic imidazolate framework ZIF-67 as an efficient co-catalyst. Appl Catal B *209*, 476-482.
